# BDH1 Attenuates Macrophage Foam Cell Formation and Inflammation in Atherosclerosis Via Modulating Histone H3K9 Acylation Balance and Activating PPAR Signaling

**DOI:** 10.1002/jbt.71141

**Published:** 2026-10-25

**Authors:** Yu Wang, Yi Wang, Qinghong Liu, Mao Tian, Bin Wang, Qiang Lin, Shuiying Huang

**Affiliations:** ^1^ Department of Cardiology Luzhou People's Hospital Luzhou Sichuan China; ^2^ Department of Pharmacy Luzhou People's Hospital Luzhou Sichuan China

**Keywords:** atherosclerosis, BDH1, histone acylation, lipid accumulation, PPAR signaling pathway

## Abstract

β‐Hydroxybutyrate dehydrogenase 1 (BDH1) has been implicated in metabolic disorders. Nevertheless, its function and underlying mechanisms in atherosclerosis (AS) remain ambiguous. A high‐fat diet‐fed apolipoprotein E‐deficient mouse model and oxidized low‐density lipoprotein (ox‐LDL)‐induced RAW264.7 macrophages were established. BDH1 expression was evaluated by immunofluorescence and Western blot. RAW264.7 cells with BDH1 overexpression or knockdown were treated with MB‐3 (acetylation inhibitor) and Ac‐CoA Synthase Inhibitor 1 (ACSS2i, β‐hydroxybutyrylation pathway inhibitor). Cell viability, lipid deposition, lipid‐related indices, inflammatory cytokines, and histone modifications were evaluated. Bioinformatics analysis was performed to screen downstream pathway of BDH1, and the peroxisome proliferator‐activated receptor (PPAR) pathway inhibitor GW9662 was applied for functional validation. Herein, BDH1 was markedly downregulated in aortic lesions and plaque macrophages from atherosclerotic mice. In ox‐LDL‐challenged RAW264.7 cells, BDH1 overexpression increased cell viability and reduced lipid accumulation, triglycerides, total cholesterol, tumor necrosis factor‐α, and histone H3 lysine 9 β‐hydroxybutyrylation (H3K9bhb) levels, while increasing interleukin‐10, and histone H3 lysine 9 acetylation (H3K9ac). Conversely, BDH1 knockdown exacerbated these pathological alterations. MB‐3 attenuated the protective effects of BDH1 overexpression and its induction of H3K9ac. ACSS2i partially rescued the deleterious effects of BDH1 knockdown, accompanied by reduced H3K9bhb and restored H3K9ac. PPAR pathway was identified as a downstream pathway of BDH1, and PPAR inhibition reversed the benefits of BDH1 overexpression. Collectively, BDH1 protects against ox‐LDL‐induced macrophage foam cell formation and inflammatory responses, at least in part by modulating histone acylation including H3K9ac and H3K9bhb, and by activating the PPAR signaling pathway.

## Graphical abstract

In atherosclerotic lesions, BDH1 expression is downregulated in plaque macrophages. In ox‐LDL‐induced RAW264.7 macrophages, BDH1 overexpression maintains H3K9 acylation homeostasis by upregulating H3K9ac and downregulating H3K9bhb, thereby increasing cell viability, reducing lipid accumulation, decreasing TNF‐α, and increasing IL‐10. Conversely, BDH1 knockdown exacerbates lipid deposition and inflammation by elevating H3K9bhb and reducing H3K9ac. Pharmacological validation shows that MB‐3 (an acetylation inhibitor) attenuates the protective effects of BDH1 overexpression, whereas ACSS2i partially rescues BDH1 knockdown‐induced injury. Furthermore, BDH1 activates the PPAR signaling pathway, and PPAR inhibition by GW9662 reverses the benefits of BDH1 overexpression. Collectively, BDH1 attenuates macrophage foam cell formation and inflammation in atherosclerosis by modulating histone H3K9 acylation balance and activating PPAR signaling.

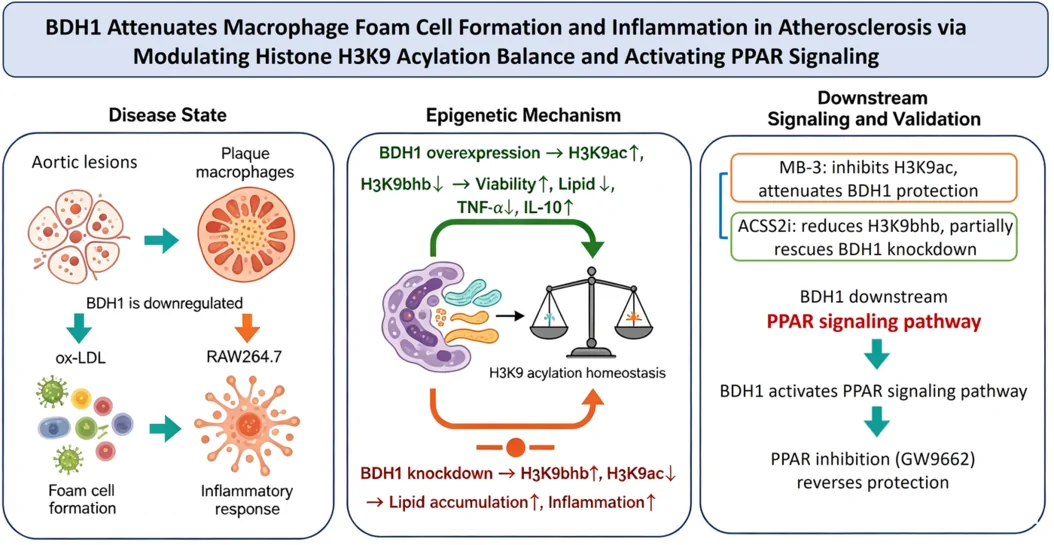

## 1 Background

Globally, atherosclerosis (AS) stands as the primary pathological foundation underlying vascular diseases; the condition's key clinical presentations—encompassing ischemic stroke, ischemic heart disease, and peripheral arterial disease—exert a severe menace to human well‐being, while generating an immense burden in terms of morbidity and mortality [1, 2]. The initiation and advancement of AS are fundamentally propelled by the persistent interplay between disordered lipid metabolism and chronic immune‐inflammatory responses within the arterial wall, collectively promoting lipid deposition, macrophage foam cell formation, and arterial wall remodeling, ultimately leading to luminal stenosis, plaque instability, and plaque rupture [3, 4]. Statin‐based lipid‐lowering therapy effectively reduces low‐density lipoprotein cholesterol (LDL‐C) levels; however, a large cohort of patients persistently face considerable residual cardiovascular risk [5]. In addition, some patients are intolerant to statins or fail to achieve optimal LDL‐C targets [6]. Therefore, further elucidation of the molecular mechanisms underlying AS and identification of novel therapeutic targets with translational potential are of substantial basic and clinical importance.

Epigenetic regulation, particularly histone post‐translational modifications, serves as a key contributor to the initiation and progression of AS [7]. Among these modifications, histone acetylation can regulate the functional states of vascular wall cells and immune cells by altering chromatin accessibility and transcriptional activity [8]. In recent years, in addition to classical histone acetylation, several emerging histone acylation modifications, including malonylation, β‐hydroxybutyrylation, lactylation, crotonylation, and succinylation, have been identified and implicated in pathological processes associated with cardiovascular diseases [9]. For example, Jiang et al. have confirmed that histone deacetylase 3 (HDAC3) serves as a promising therapeutic target for AS [10]. SLC22A6‐dependent lactate accumulation in histone H3 lysine 9 (H3K9) exacerbates endothelial dysfunction and AS [11]. Nevertheless, the precise biological functions of distinct histone acylation modifications and their regulatory mechanisms in AS remain to be further elucidated.

β‐Hydroxybutyrate dehydrogenase 1 (BDH1) is a key rate‐limiting enzyme in ketone body metabolism. It primarily catalyzes the reversible interconversion between β‐hydroxybutyrate and acetoacetate, thereby maintaining ketone body homeostasis [12]. In recent years, the biological functions of BDH1 in diverse pathological conditions, including metabolic diseases, cancer, and cardiomyopathy, have attracted increasing attention [12, 13, 14]. Previous studies have shown that BDH1 overexpression alleviates diabetic cardiomyopathy by suppressing lipocalin‐2 (LCN2) transcriptional activation mediated by histone H3 lysine 9 β‐hydroxybutyrylation (H3K9bhb) [15]. Lin et al. have documented a downregulation of BDH1 expression in patients with diabetes mellitus and AS, high‐glucose‐treated macrophages, and diabetic apolipoprotein E‐deficient (ApoE^−^/^−^) mice; adeno‐associated virus (AAV)‐mediated BDH1 overexpression mitigates aortic plaque formation in diabetic ApoE^−^/^−^ mice, potentially by promoting ketone body metabolism and improving lipid metabolic abnormalities [16]. However, the regulatory role of BDH1 in histone acylation modifications during AS and its molecular mechanisms driving this process are still inadequately characterized.

This study is dedicated to elucidating the role of BDH1 in the oxidized low‐density lipoprotein (ox‐LDL)‐triggered foam cell formation within RAW264.7 macrophages, with a key focus on determining whether BDH1 modulates macrophage lipid accumulation and inflammatory responses through its regulatory action on histone acyl modification. Additionally, through bioinformatics‐based screening, this research has identified the peroxisome proliferator‐activated receptor (PPAR) signaling pathway as a downstream cascade of BDH1 in AS, deepening the understanding of AS pathogenesis and offering promising novel targets for the prevention and treatment of AS.

## 2 Materials and Methods

### 2.1 Construction of the In Vivo Model

All animal experiments were sanctioned by the Institutional Animal Care and Use Committee of Southwest Medical University (No. 20221109‐012). Male C57BL/6 mice and ApoE^−^/^−^ congenic mice (6–8 weeks old, 18–20 g body weight) were purchased from SPF Biotechnology Co. Ltd (Beijing, China). All animals were acclimatized for 1 week under specific‐pathogen‐free (SPF) barrier conditions at a constant temperature of (22 ± 2)°C and relative humidity of 50%–60% under a 12 h light/dark cycle, and baseline fasting blood glucose (FBG) was measured with a glucometer at the end of the acclimatization period. C57BL/6 mice were fed a standard rodent chow and served as the Control group, whereas ApoE^−^/^−^ mice were fed a high‐fat diet (D12079B, Special Diet Services Ltd., Essex, UK) for 12 consecutive weeks to establish the AS model [17, 18]. FBG was monitored every 4 weeks during the modeling period, and peripheral blood and aortic tissue samples were collected at the experimental endpoint for biochemical and histopathological analyses. Mice were anesthetized via isoflurane inhalation, with an induction concentration of 3%–4% and a maintenance concentration of 1.5%–2%. Subsequently, the mice were sacrificed by cervical dislocation.

### 2.2 Enzyme‐Linked Immunosorbent Assay (ELISA)

Serum levels of total cholesterol (TC), triglycerides (TG), high‐density lipoprotein cholesterol (HDL‐C), LDL‐C, interleukin‐10 (IL‐10), and tumor necrosis factor‐α (TNF‐α) were assessed using the corresponding ELISA kits (Esebio, Shanghai, China). The required microplate strips were removed from the foil pouch after equilibration at room temperature for 20 min, and the remaining strips were sealed and stored at 4°C. Standard, sample, and blank wells were designated on each plate: 50 μL of standard at different concentrations was added to the standard wells; 10 μL of the test sample and 40 μL of sample diluent were added to the sample wells; no reagent was added to the blank wells. Except for the blank wells, 100 μL of horseradish peroxidase (HRP)‐conjugated detection antibody was added to each well, and the plate was sealed and incubated at 37°C for 60 min. The wells were then emptied and blotted dry on absorbent paper, filled with wash buffer for 1 min, and washed five times. Subsequently, 50 μL of substrates A and B was added to each well and the plate was incubated at 37°C in the dark for 15 min, followed by 50 μL of stop solution. The optical density (OD) of each well was measured at 450 nm within 15 min.

### 2.3 Hematoxylin–Eosin (H&E) Staining

Mouse aortic tissues were fixed in 4% paraformaldehyde, rinsed with running water the following day, and dehydrated through a graded ethanol series (50%–70%–80%–90%–95%–100%). The tissues were then cleared sequentially in a 1:1 mixture of anhydrous ethanol and xylene followed by pure xylene, and embedded in paraffin with two changes of paraffin to ensure complete removal of residual xylene. After trimming, 4‐μm‐thick serial sections were cut, flattened in a water bath, and dried. The sections were baked at 60°C for 1–2 h, deparaffinized in xylene, and rehydrated through graded ethanol. They were stained with hematoxylin for 10 min, rinsed in running water, differentiated in 0.7% hydrochloric acid–ethanol for several seconds, and returned to blue in running water for approximately 15 min. The sections were then processed through 95% ethanol, alcoholic eosin staining, graded ethanol dehydration, and clearing in carbolic xylene and xylene, and finally mounted with neutral balsam. Histomorphological changes were observed and imaged under a light microscope.

### 2.4 Oil Red O Staining for Lipid Deposition

Tissue sections were brought to room temperature, washed with distilled water to remove the embedding medium, and rinsed in 60% isopropanol for 2 min. The sections were then immersed in 0.5% Oil Red O solution (prepared in propylene glycol) for 15–30 min, differentiated in 60% isopropanol, and immediately washed with water. Nuclei were counterstained with hematoxylin (strictly controlled to <1 min), differentiated in acid–ethanol for 1–5 s, and returned to blue in running water. The sections were mounted with glycerin gelatin (an aqueous mounting medium) and imaged under a microscope in multiple fields. For cytospin preparations, cell suspensions were evenly spread onto poly‐l‐lysine‐coated slides, air‐dried at room temperature, fixed with paraformaldehyde for 5 min, and stored at –20°C until use.

### 2.5 Immunofluorescence (IF) Staining

The paraffin sections underwent a sequential processing: first, baking at 60°C for 60 min, followed by deparaffinization in xylene, rehydration via graded ethanol, and a 5 min immersion in distilled water. Subsequently, antigen retrieval was conducted in pH: 9.0 Ethylene Diamine Tetraacetic Acid alkaline retrieval solution—microwave heating at high power for 2.5 min until boiling, then low‐power maintenance for 20 min, with subsequent cooling at room temperature for 20 min. Endogenous peroxidase activity was suppressed by treating the sections with 3% hydrogen peroxide at room temperature in the dark for 10 min. After washing with phosphate buffered saline (PBS), the sections were incubated with goat serum blocking solution at room temperature in the dark for 30 min. Primary antibodies against BDH1 and F4/80 were then applied and incubated at 4°C overnight, followed by three PBS washes. HRP‐conjugated secondary antibodies were applied at room temperature in the dark for 30 min, and after PBS washing, tyramide signal amplification (TSA) fluorophore reaction solution was added and incubated at room temperature in the dark for 10 min. For multiplex staining, antibody stripping was performed by microwave heating in antigen retrieval solution between rounds, after which the blocking, primary antibody, secondary antibody, and TSA reaction steps were repeated. Following the final TSA reaction, nuclei were counterstained with 4',6‐diamidino‐2‐phenylindole for 5 min, and the sections were mounted with an anti‐fluorescence quenching mounting medium and imaged using a confocal laser scanning microscope.

### 2.6 Cell Grouping and Treatment

Murine monocyte‐macrophage cells (RAW264.7), sourced from iCell Bioscience (Shanghai, China), were cultured in high‐glucose Dulbecco's modified Eagle's medium (DMEM) with the addition of 10% fetal bovine serum (FBS) and 1% penicillin‐streptomycin. The cells were cultured in a humidified incubator at 37°C with 5% CO_2_. The groups were designated as follows: Control, ox‐LDL, si‐NC, si‐BDH1, oe‐NC, oe‐BDH1, oe‐BDH1 + MB‐3, si‐BDH1 + ACSS2i, and oe‐BDH1 + GW9662. To establish the foam cell model, cells in other groups were induced with 100 μg/mL ox‐LDL for 24 h [19], excluding the Control group.

For genetic manipulation, BDH1 knockdown and overexpression were achieved by transfecting cells with BDH1‐specific small interfering RNA (si‐BDH1) or BDH1‐overexpressing plasmids, respectively, using Lipofectamine 3000 (Thermo Fisher Scientific, Waltham, MA, USA) according to the manufacturer's protocol. Corresponding negative controls (si‐NC and oe‐NC) were employed. To investigate specific signaling pathways, pharmacological inhibitors were applied: MB‐3 (50 μM) was used to inhibit BDH1‐mediated acetylation [20]; Ac‐CoA Synthase Inhibitor 1 (ACSS2i, 10 μM) was administered for 6 h to block the β‐hydroxybutyrylation pathway [21]; and PPAR signaling was suppressed by pre‐treating cells with 1 μM GW9662 (dissolved in DMSO) for 2 h [22].

### 2.7 Cell Counting Kit (CCK)−8 Assay

A 100 μL volume of cell suspension was seeded into a 96‐well plate and pre‐incubated for 24 h to achieve a cell confluence of approximately 80%. The culture medium was then replaced with a maintenance medium, and the cells were further incubated for another 24 h. Following the removal of the culture medium, the cells underwent two washes with Dulbecco's phosphate‐buffered saline (DPBS). Next, each well received 90 μL of maintenance medium and 10 μL of CCK‐8 solution, followed by an additional 2 h incubation. The absorbance at 450 nm was then quantified via a microplate reader, with measurements collected at 1 h intervals to identify the optimal reading.

#### 2.7.1 Real‐Time Quantitative Polymerase Chain Reaction (RT‐qPCR)

Total RNA was isolated from cells using the TRIZOL reagent, followed by reverse transcription to generate cDNA. RT‐qPCR was conducted on an ABI 7500 quantitative PCR system (Applied Biosystems, NY, USA). The thermal cycling protocol was established as: pre‐denaturation at 95°C for 30 s; followed by 40 cycles of denaturation at 95°C for 10 s and annealing/extension at 60°C for 30 s. The relative expression levels were quantified via the 2^‐ΔΔCt^ method. Primers, synthesized by Sangon Biotech Co. Ltd. (Shanghai, China), had their sequences fully documented in Table S1.

### 2.8 Western Blot

Total proteins were extracted from aortic tissues and cellular samples. Subsequently, 5× loading buffer was added, and the mixture was denatured by boiling for 10 min. The samples were then subjected to sodium dodecyl sulfate polyacrylamide gel electrophoresis and transferred onto a polyvinylidene fluoride‌ membrane under conditions of 4°C for 2 h. After blocking with 5% non‐fat milk in Tris buffered saline with Tween‐20 (TBST) at room temperature for 2 h, the membrane was incubated with primary antibodies (1/1000) targeting BDH1 (ab193156, Abcam, Cambridge, Britain), H3K9ac (ab32129, Abcam), H3K9bhb (PTM‐1250RM, PTM Biolabs Inc, Hangzhou, China), Histone H3 (H3, ab1791, Abcam), PPARγ (ab45036, Abcam), and PPARα (ab61182, Abcam) overnight at 4°C. Following TBST‐based washing of the membrane, incubation with goat anti‐rabbit secondary antibody (1/5000, ab6721, Abcam) was conducted at room temperature for 1 h. Chemiluminescence detection and exposure were performed, and the resulting data were analyzed using ImageJ software.

### 2.9 Bioinformatic Analysis and Pathway Screening

To identify potential signaling pathways regulated by BDH1 in AS, we employed a bioinformatics approach. First, AS‐associated genes were retrieved from the GeneCards database (https://www.genecards.org/) using “Atherosclerosis” as the keyword. Second, the top 2000 genes co‐expressed with BDH1 were screened using the CoXPresDB database (https://coxpresdb.jp/). The intersection of these two gene sets was visualized using a Venn diagram. Subsequently, Kyoto Encyclopedia of Genes and Genomes (KEGG) pathway enrichment analysis was performed on the intersecting genes to identify significantly enriched signaling pathways.

### 2.10 Statistical Analysis

All data underwent analysis with GraphPad Prism 8.0 and were represented as mean ± standard deviation. For pairwise comparisons, the independent samples t‐test was applied, whereas one‐way ANOVA combined with Tukey's post hoc test was executed for multi‐group analyses. Statistical significance was established at a *P*‐value of less than 0.05.

## 3 Results

### 3.1 Establishment and Validation of the AS Mouse Model

To evaluate the successful construction of the AS model, we conducted a comprehensive assessment comparing the Control group and the AS model group. Regarding metabolic parameters, as illustrated in Figure 1A, the AS model group exhibited significantly elevated FBG levels compared to the Control group. Furthermore, serum lipid analysis revealed a marked dyslipidemia in the AS group, characterized by significantly increased levels of TC, TG, and LDL‐C, concomitant with a significant reduction in HDL‐C (Figure 1B).

**Figure 1:**
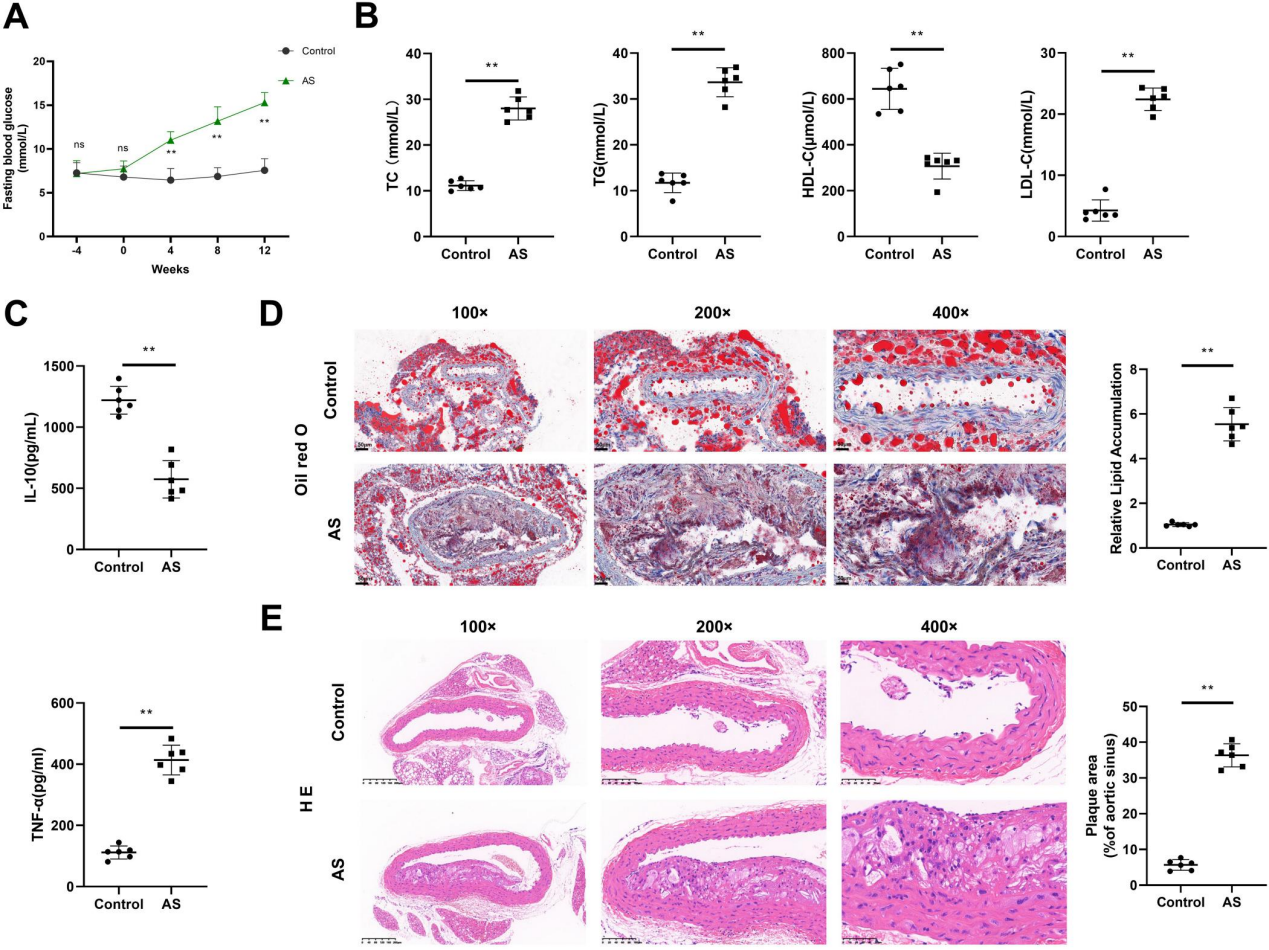
Establishment of the atherosclerosis (AS) mouse model. (A) Measurement of fasting blood glucose levels in mice. (B) Analysis of serum lipid profiles, including triglycerides (TG), total cholesterol (TC), high‐density lipoprotein cholesterol (HDL‐C), and low‐density lipoprotein cholesterol (LDL‐C) by Enzyme‐Linked Immunosorbent Assay (ELISA). (C) Detection of serum inflammatory cytokines, specifically interleukin‐10 (IL‐10) and tumor necrosis factor‐α (TNF‐α) by ELISA. (D) Representative photomicrographs and quantitative analysis of lipid accumulation in aortic tissues stained with Oil Red O. Scale bars: 200 μm (100×), 100 μm (200×), and 50 μm (400×). (E) Representative images of Hematoxylin–Eosin (H&E) staining and quantitative analysis of plaque area (% of aortic sinus) in aortic tissues. Scale bars: 200 μm (100×), 100 μm (200×), and 50 μm (400×). Data are presented as mean ± standard deviation (SD). *n* ≥ 6 mice per group. **p < 0.01.

In terms of the inflammatory response, the AS model group demonstrated a distinct inflammatory imbalance (Figure 1C). Specifically, serum levels of TNF‐α were significantly elevated, whereas IL‐10 was significantly reduced compared to the Control group.

Pathological examination of the aorta further confirmed the model's validity. Oil Red O staining showed that while the aortic intima of the Control group remained smooth without visible lipid plaques, the AS model group presented with evident lipid deposition within the vascular wall (Figure 1D). Consistently, H&E staining indicated that the AS group suffered from severe aortic injury, manifesting as significant intimal thickening and substantial inflammatory cell infiltration (Figure 1E). Collectively, these findings definitively validate the successful construction of the AS mouse model.

### 3.2 Downregulation of BDH1 Expression in Atherosclerotic Lesions

To elucidate the expression profile of BDH1 during the pathogenesis of AS, we evaluated its protein levels in the aortic tissues of Control and AS model mice. As shown in Figure 2A, Western blot analysis revealed a marked reduction in BDH1 protein expression in the aortas of AS model mice compared to the Control group. Consistently, IF demonstrated that while the Control group exhibited robust BDH1 fluorescence signals predominantly localized in the vascular intima and media, the AS model group displayed significantly attenuated signals (Figure 2B). Furthermore, to determine the specific expression of BDH1 within plaque‐resident macrophages, we performed co‐localization analysis of BDH1 and the macrophage marker F4/80. As depicted in Figure 2C, in the Control group, distinct green fluorescence signals corresponding to BDH1 were observed within the lesion area, which largely colocalized with the red fluorescence signals of F4/80‐positive macrophages, appearing as yellow regions in the merged images. This indicated that BDH1 was primarily expressed in macrophages under pathological conditions. In contrast, in the AS group, both the green fluorescence intensity of BDH1 and the red fluorescence intensity of F4/80 were markedly diminished compared to the Control group.

**Figure 2:**
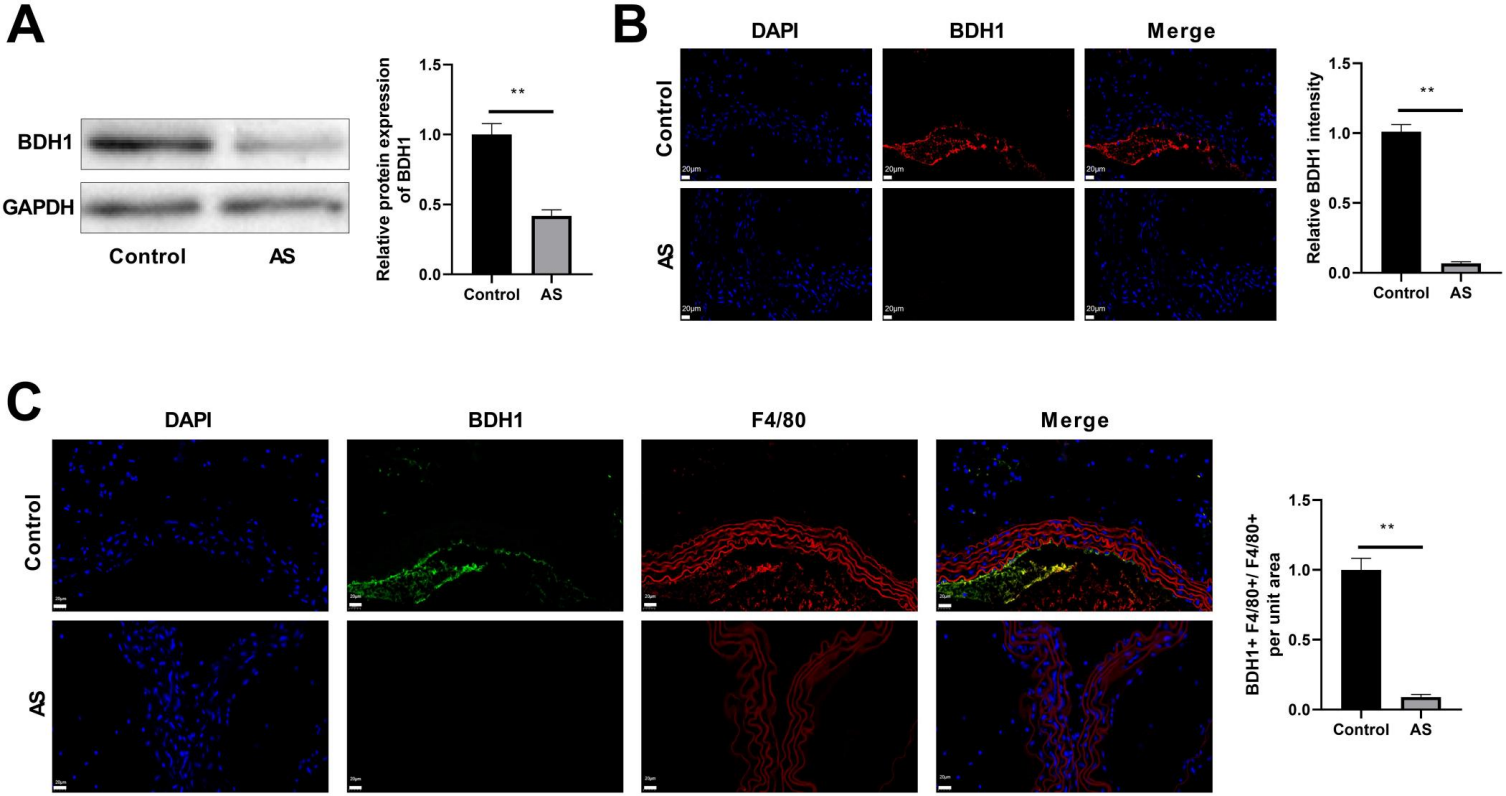
Downregulation of β‐Hydroxybutyrate dehydrogenase 1 (BDH1) expression in atherosclerotic lesions. (A) Representative Western blot bands and quantification of BDH1 protein levels in aortic tissues. (B) Representative immunofluorescence (IF) images showing BDH1 expression in aortic sections. Scale bars: 20 μm (400×). The mean fluorescence intensity of BDH1 was quantified using ImageJ software. (C) Co‐localization of BDH1 (green) and the macrophage marker F4/80 (red) in atherosclerotic plaques. Co‐localization was quantified using ImageJ. Scale bars: 20 μm (400×). Data are presented as mean ± SD. *n* ≥ 6 mice per group. **p < 0.01. Representative Western blot bands are from three independent experiments, and densitometric quantification was performed using all independent experiments.

#### 3.2.1 BDH1 Regulates ox‐LDL‐Induced RAW264.7 Macrophage Function Via Histone Acylation Modifications

To investigate the role of BDH1 in macrophage foam cell formation and its regulatory mechanism through acylation modifications, BDH1 overexpression and knockdown models were established in RAW264.7 cells. Briefly, a BDH1 overexpression plasmid and BDH1‐specific siRNA were transfected into in vitro‐cultured RAW264.7 cells, respectively, and the transfection efficiency was verified by qPCR analysis of BDH1 mRNA expression. As shown in Figure 3A, the expression of BDH1 exhibited no significant difference when comparing the ox‐LDL group with the ox‐LDL+oe‐NC and ox‐LDL+si‐NC groups; in ox‐LDL induced RAW264.7 cells, BDH1 mRNA expression was significantly increased in the BDH1 overexpression group and markedly decreased in the BDH1 knockdown group compared with the negative control group, indicating that the overexpression and knockdown constructs were successfully established and that the transfection efficiency met the requirements for subsequent experiments.

**Figure 3:**
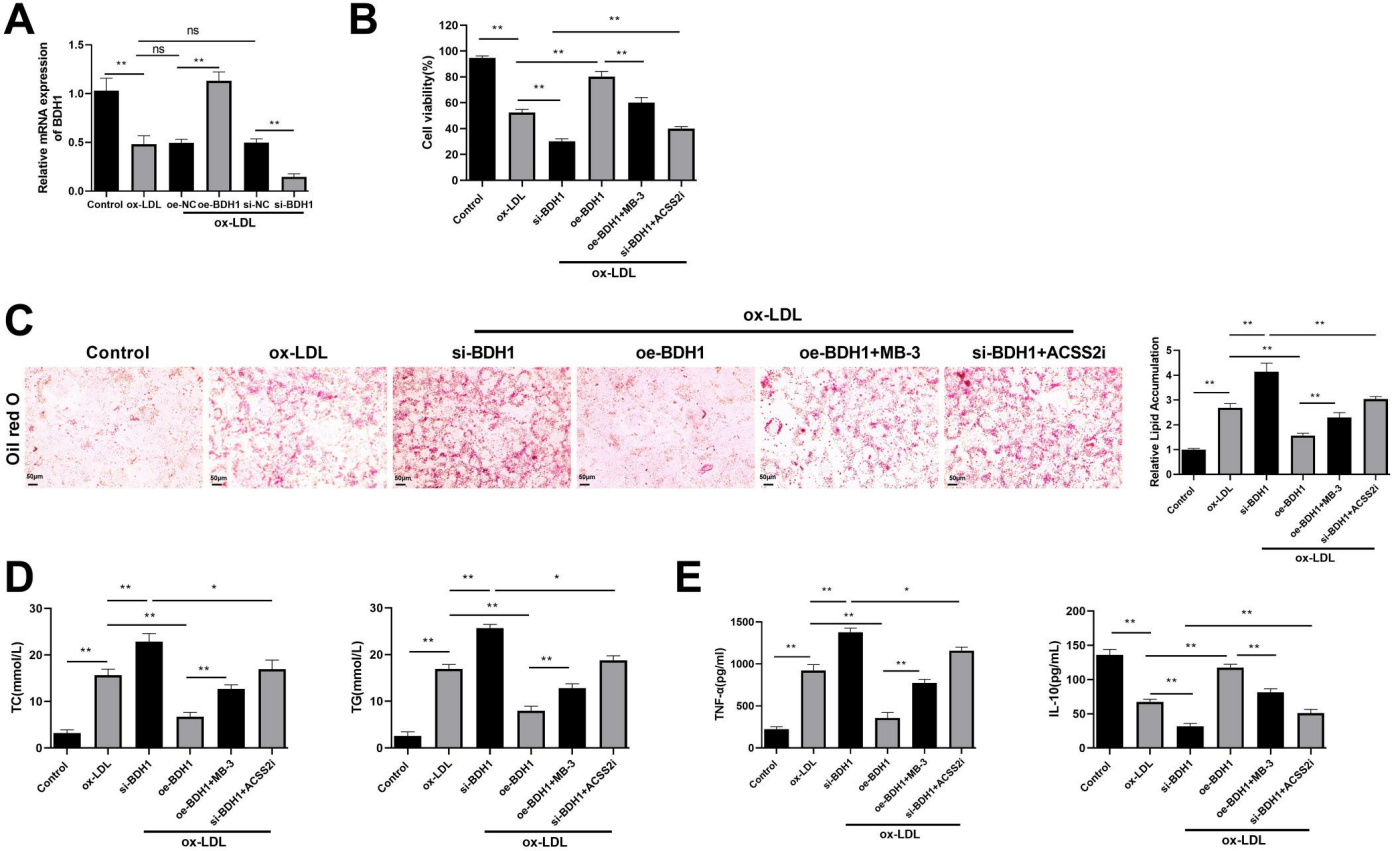
Effects of BDH1 modulation on lipid accumulation and inflammation in RAW264.7 cells. (A) Verification of BDH1 overexpression and silencing efficiency in RAW264.7 cells using Real‐Time Quantitative Polymerase Chain Reaction (RT‐qPCR). (B) Cell viability was assessed using the Cell Counting Kit (CCK)−8 assay. (C) Intracellular lipid accumulation was evaluated and quantified by Oil Red O staining. Scale bars: 50 μm (400×). (D) Levels of intracellular lipids (TG, TC) were evaluated by ELISA. (E) Secretion levels of inflammatory cytokines (TNF‐α and IL‐10) were detected by ELISA. Data are presented as mean ± SD. *n* ≥ 3 independent biological replicates, each with three technical replicates. **p < 0.01, *p < 0.05, ns: not significant.

CCK‐8 assay showed that cell viability was significantly reduced in the ox‐LDL‐induced model group compared with the normal Control group. Compared with the ox‐LDL group, BDH1 knockdown further decreased cell viability, suggesting that BDH1 silencing aggravated ox‐LDL‐induced cell injury, whereas BDH1 overexpression significantly restored cell viability, indicating that BDH1 overexpression alleviated the ox‐LDL‐induced decline in cell viability. Co‐treatment with MB‐3 (an acetylation inhibitor) significantly reduced cell viability in the BDH1 overexpression group, suggesting that MB‐3 partially reversed the protective effect of BDH1 overexpression. Moreover, the ACSS2 inhibitor significantly increased cell viability in the BDH1 knockdown group, indicating that suppression of the β‐hydroxybutyrylation pathway partially rescued the cell injury caused by BDH1 silencing (Figure 3B).

Oil Red O staining revealed no obvious red‐stained lipid droplets in the cytoplasm of normal Control cells, whereas the ox‐LDL‐induced model group exhibited abundant diffuse red lipid droplets, confirming successful foam cell model establishment. Compared with the ox‐LDL group, BDH1 knockdown further increased the number and size of lipid droplets and significantly enlarged the red‐stained area, whereas BDH1 overexpression markedly reduced the number of lipid droplets and the red‐stained area. Co‐treatment with MB‐3 aggravated lipid deposition compared with BDH1 overexpression alone, whereas the ACSS2 inhibitor significantly attenuated lipid deposition compared with BDH1 knockdown alone (Figure 3C).

Lipid‐related indices analyzed via ELISA revealed distinct differences between groups: relative to the Control group, the ox‐LDL model group exhibited marked elevations in TG and TC. Notably, BDH1 knockdown amplified the increases in TG and TC compared with the ox‐LDL group, while BDH1 overexpression conversely attenuated the elevations of TG and TC levels. MB‐3 treatment significantly attenuated the BDH1 overexpression‐induced improvement in these lipid indices, whereas the ACSS2 inhibitor significantly reversed the BDH1 knockdown‐induced deterioration of lipid profiles (Figure 3D). In addition, compared with the Control group, the pro‐inflammatory cytokine TNF‐α was significantly increased and the anti‐inflammatory cytokine IL‐10 was significantly decreased in the ox‐LDL group. Compared with the ox‐LDL group, BDH1 knockdown further elevated TNF‐α and further reduced IL‐10, whereas BDH1 overexpression significantly decreased TNF‐α and increased IL‐10. Co‐treatment with MB‐3 significantly increased TNF‐α and decreased IL‐10 compared with BDH1 overexpression alone, suggesting that acetylation inhibition reversed the anti‐inflammatory effect of BDH1 overexpression. The ACSS2i significantly reduced TNF‐α and increased IL‐10 compared with BDH1 knockdown alone, indicating that suppression of β‐hydroxybutyrylation partially rescued the pro‐inflammatory effect of BDH1 silencing (Figure 3E).

To further verify the regulatory effect of BDH1 on histone post‐translational modifications, the modification levels of H3K9ac (acetylation) and H3K9bhb (β‐hydroxybutyrylation) were determined in each group. Figure 4A,B illustrated that, relative to the ox‐LDL group, BDH1 expression was markedly downregulated in the BDH1 knockdown group and prominently upregulated in the BDH1 overexpression group. BDH1 expression levels in the oe‐BDH1 + MB‐3 and si‐BDH1 + ACSS2i groups demonstrated no significant differences from those in the oe‐BDH1 and si‐BDH1 groups, respectively, indicating that neither MB‐3 nor the ACSS2i affected BDH1 expression itself.

**Figure 4:**
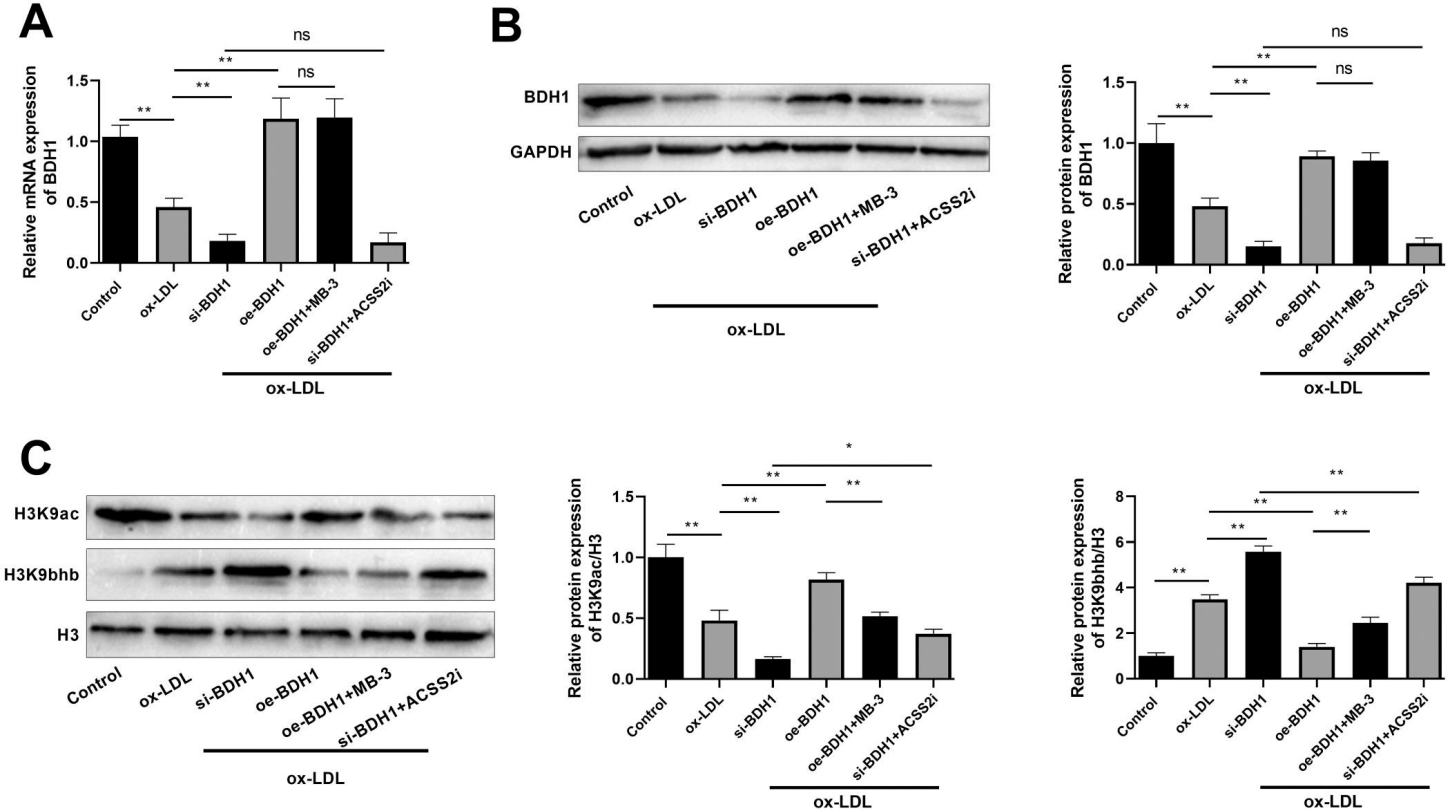
BDH1 regulates histone acetylation and β‐hydroxybutyrylation levels. (A, B) mRNA and protein expression levels of BDH1 were detected by RT‐qPCR and Western blot, respectively. (C) Western blot analysis of histone H3 lysine 9 acetylation (H3K9ac) and histone H3 lysine 9 β‐hydroxybutyrylation (H3K9bhb) levels. Data are presented as mean ± SD. *n* ≥ 3 independent biological replicates, each with three technical replicates. **p < 0.01, *p < 0.05, ns: not significant. Representative Western blot bands are from three independent experiments, and densitometric quantification was performed using all independent experiments.

Western blot analysis was subsequently conducted to assess the levels of H3K9ac and H3K9bhb (Figure 4C). BDH1 overexpression significantly increased H3K9ac levels, whereas BDH1 silencing markedly decreased them, suggesting that BDH1 is associated with the maintenance of H3K9ac. Treatment with MB‐3 effectively abolished the BDH1 overexpression‐induced increase in H3K9ac. Conversely, inhibiting the ACSS2‐mediated pathway with ACSS2i partially restored the reduced H3K9ac levels caused by BDH1 knockdown. Regarding H3K9bhb, an inverse trend was observed. BDH1 overexpression significantly suppressed H3K9bhb levels, while BDH1 silencing notably elevated them. Importantly, the reduction in H3K9bhb induced by BDH1 overexpression was reversed by MB‐3 treatment. Furthermore, the application of ACSS2i effectively blocked the upregulation of H3K9bhb triggered by BDH1 silencing. Collectively, these findings demonstrate that BDH1 overexpression was associated with increased H3K9ac and decreased H3K9bhb levels, whereas BDH1 knockdown showed the opposite trend. These changes were accompanied by alterations in cell viability, lipid accumulation, and inflammatory responses. However, given that MB‐3 and ACSS2i are not site‐specific inhibitors, these data indicate an association between BDH1‐mediated phenotypes and changes in H3K9 acylation, rather than establishing a direct causal role for H3K9 modifications alone.

#### 3.2.2 Bioinformatic Screening Identifies the PPAR Signaling Pathway as a Potential Downstream Target of BDH1

To elucidate the molecular mechanisms underlying the protective role of BDH1 in AS, we performed a bioinformatic screening for potential downstream targets. We identified 490 intersecting genes between the top 2000 BDH1 co‐expressed genes and AS‐associated genes (Figure 5A). KEGG pathway enrichment analysis of these 490 intersecting genes revealed that the metabolic pathways, Carbon metabolism, and PPAR signaling pathway were significantly enriched (Figure 5B).

**Figure 5:**
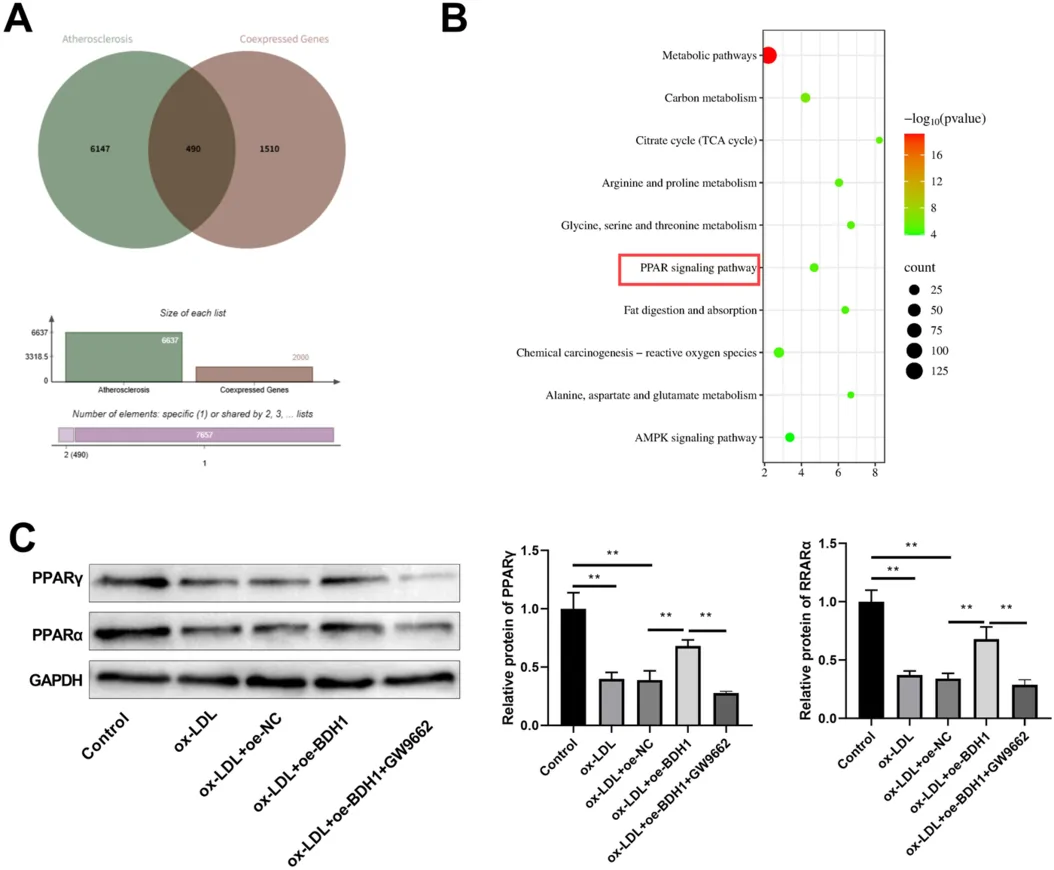
Bioinformatic analysis and verification of the peroxisome proliferator‐activated receptor (PPAR) signaling pathway. (A) Venn diagram showing the intersection of the top 2000 BDH1 co‐expressed genes and AS‐associated genes. (B) Kyoto Encyclopedia of Genes and Genomes (KEGG) pathway enrichment analysis of the intersecting genes. (C) Western blot analysis of protein expression levels associated with the PPAR signaling pathway. Data are presented as mean ± SD. *n* ≥ 3 independent biological replicates, each with three technical replicates. **p < 0.01. Representative Western blot bands are from three independent experiments, and densitometric quantification was performed using all independent experiments.

To validate these findings, we examined the protein expression of key PPAR signaling components, PPARγ and PPARα, using Western blot (Figure 5C). Compared with the Control group, ox‐LDL significantly suppressed PPARγ and PPARα levels, indicating pathway suppression during AS. Notably, while transfection with empty vector (oe‐NC) had no effect, BDH1 overexpression significantly reversed the ox‐LDL‐induced reduction in PPARγ and PPARα levels. Furthermore, treatment with GW9662, a specific PPAR antagonist, abolished the upregulation of PPARγ and PPARα mediated by BDH1 overexpression. In aggregate, these data suggest that BDH1 exerts its protective effects by activating the PPAR signaling pathway.

#### 3.2.3 BDH1 Alleviates ox‐LDL‐Induced RAW264.7 Macrophage Injury and Foam Cell Formation Through the PPAR Signaling Pathway

To determine whether BDH1 exerts its anti‐atherosclerotic effects through the PPAR signaling pathway, the PPAR pathway inhibitor GW9662 was introduced into an ox‐LDL‐induced RAW264.7 macrophage model. The experimental groups included a Control group, an ox‐LDL+oe‐NC group, an ox‐LDL+oe‐BDH1 group, and an ox‐LDL+oe‐BDH1 + GW9662 group.

CCK‐8 assay indicated that cell viability was significantly decreased in the ox‐LDL+oe‐NC group relative to the Control group, indicating that ox‐LDL exposure induced injury in RAW264.7 macrophages. Compared with the ox‐LDL+oe‐NC group, cell viability was significantly promoted in the ox‐LDL+oe‐BDH1 group, suggesting that BDH1 overexpression partially alleviated the ox‐LDL‐induced reduction in cell viability. However, cell viability was significantly decreased in the ox‐LDL+oe‐BDH1 + GW9662 group in comparison with the ox‐LDL+oe‐BDH1 group (Figure 6A), indicating that GW9662 partially attenuated the cytoprotective effect of BDH1 overexpression.

**Figure 6:**
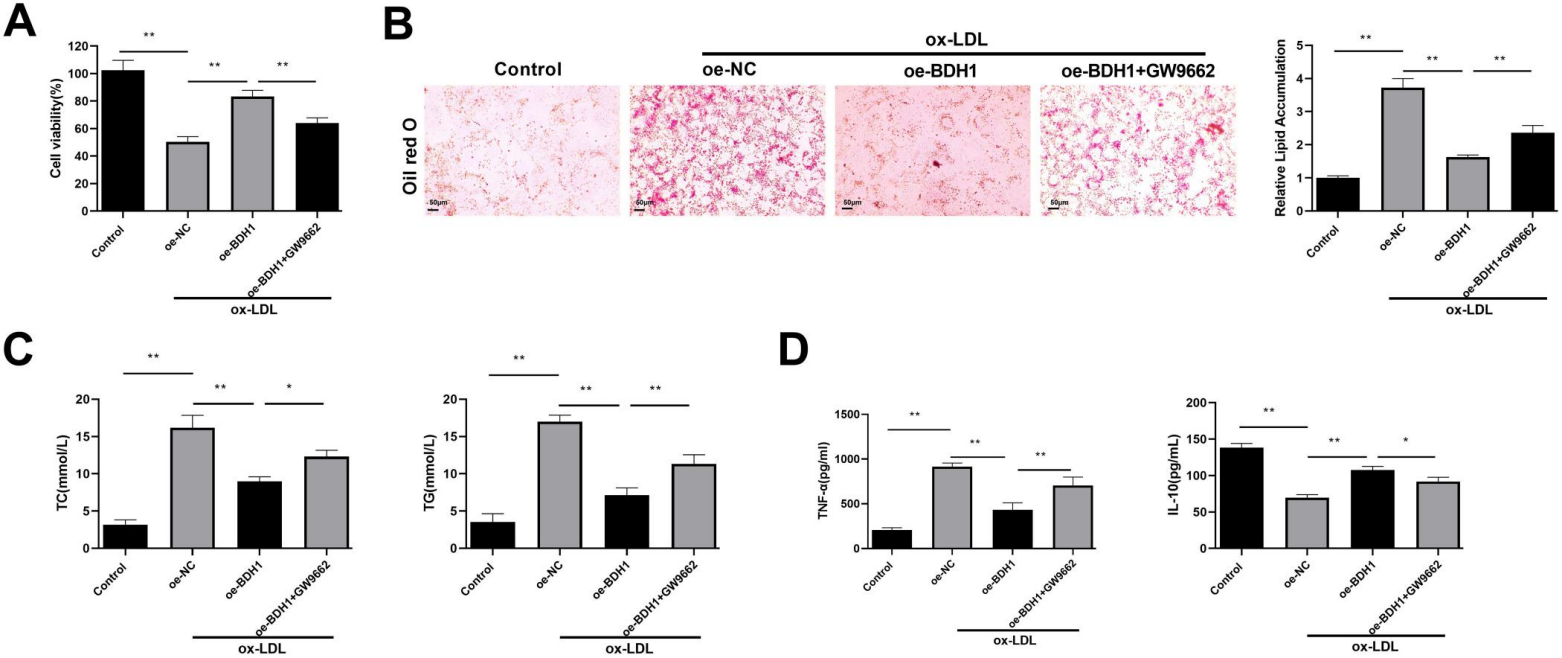
Functional rescue experiments in vitro. (A) Cell viability was determined by CCK‐8 assay. (B) Lipid deposition was assessed and quantified by Oil Red O staining. Scale bars: 50 μm (400×). (C) Intracellular lipid levels (TG, TC) were measured by ELISA. (D) Expression levels of inflammatory cytokines (TNF‐α and IL‐10) were detected by ELISA. Data are presented as mean ± SD. *n* ≥ 3 independent biological replicates, each with three technical replicates. **p < 0.01, *p < 0.05.

Oil Red O staining demonstrated that cells in the Control group displayed normal morphology without apparent cytoplasmic lipid droplet accumulation. In contrast, abundant diffuse red‐stained lipid droplets and typical foam cell‐like morphology were observed in the ox‐LDL+oe‐NC group. In contrast to the ox‐LDL+oe‐NC group, the number of intracellular lipid droplets and the red‐stained area were significantly reduced in the ox‐LDL+oe‐BDH1 group, indicating that BDH1 overexpression inhibited ox‐LDL‐induced lipid accumulation and foam cell formation. Following GW9662 treatment, intracellular lipid deposition and the red‐stained area were significantly increased in the ox‐LDL+oe‐BDH1 + GW9662 group in contrast with the ox‐LDL+oe‐BDH1 group, suggesting that PPAR pathway inhibition partially reversed the inhibitory effect of BDH1 overexpression on lipid accumulation (Figure 6B).

ELISA analysis revealed that, relative to the Control group, the ox‐LDL+oe‐NC group showed marked elevations in TG and TC. Additionally, the pro‐inflammatory cytokine TNF‐α level was significantly upregulated, while the anti‐inflammatory cytokine IL‐10 level was markedly downregulated. Notably, BDH1 overexpression, in contrast to the ox‐LDL+oe‐NC group, significantly attenuated the levels of TG, TC, and TNF‐α, alongside a marked increase in IL‐10 levels (Figure 6C,D). These findings indicated that BDH1 overexpression ameliorated ox‐LDL‐induced lipid metabolic disturbances and exerted anti‐inflammatory effects. Notably, compared with the ox‐LDL+oe‐BDH1 group, the ox‐LDL+oe‐BDH1 + GW9662 group showed significantly increased TG, TC, and TNF‐α levels, whereas IL‐10 levels were significantly decreased (Figure 6C,D), further suggesting that GW9662 partially reversed the beneficial effects of BDH1 overexpression on lipid metabolic abnormalities and inflammatory responses.

## 4 Discussion

AS serves as the principal pathological foundation for cardiovascular diseases—encompassing ischemic heart disease and stroke—and constitutes a grave threat to human well‐being [23, 24]. In the present study, BDH1 expression was downregulated in atherosclerotic lesions. Functional experiments demonstrated that BDH1 overexpression alleviated ox‐LDL‐induced reductions in macrophage viability, lipid accumulation, and inflammatory responses, whereas BDH1 knockdown exacerbated these pathological alterations. Mechanistically, BDH1 exerted protective effects by maintaining histone acylation homeostasis through upregulation of H3K9ac and downregulation of H3K9bhb, in conjunction with activation of the PPAR signaling pathway. Inhibition of histone acetylation or blockade of PPAR signaling attenuated the protective effects of BDH1 overexpression, whereas inhibition of β‐hydroxybutyrylation partially ameliorated the cellular injury induced by BDH1 knockdown.

Histone post‐translational modifications play important regulatory roles in lipid metabolic dysregulation, cellular phenotypic plasticity, and inflammatory responses during AS [7, 25]. In recent years, histone acylation modifications have received increasing attention in the context of AS pathogenesis and progression [9, 26]. Previous studies have shown that histone deacetylase 6 (HDAC6) deficiency enhances signal transducer and activator of transcription 3 lysine 685 acetylation (STAT3‐K685ac), upregulates the expression of the scavenger receptors CD36 and SR‐A, and promotes macrophage cholesterol uptake, thereby aggravating AS [27]. HDAC3 has also been reported to promote AS progression by activating the endothelial NLRP3 inflammasome and suppressing the acetylation of specificity protein 1 (SP1) [28]. In addition, knockdown of acetyl‐CoA synthetase short‐chain family member 2 (ACSS2) suppresses AS progression by reducing histone H3 lysine 9 crotonylation (H3K9cr) [29]. Herein, BDH1 overexpression significantly increased H3K9ac and decreased H3K9bhb, whereas BDH1 knockdown exerted opposite effects. The acetylation inhibitor MB‐3 reversed the BDH1 overexpression‐induced increase in H3K9ac and attenuated its beneficial effects on cellular injury, lipid accumulation, and inflammatory responses. Conversely, ACSS2 inhibition suppressed the increase in H3K9bhb induced by BDH1 knockdown and partially alleviated the associated cellular injury. Collectively, BDH1 may regulate macrophage function at least in part by modulating histone acylation, including H3K9ac and H3K9bhb.

PPARs are ligand‐activated members of the nuclear receptor superfamily and comprise three isoforms: PPARα, PPARβ/δ, and PPARγ [30]. By regulating lipid metabolism, inflammatory responses, and redox homeostasis, PPARs play essential roles in AS initiation and progression [31]. PPARα agonists reduce TG levels and possess anti‐inflammatory properties, making them potential therapeutic candidates for reducing residual cardiovascular risk [32]. In macrophages, activation of PPARγ signaling promotes cholesterol efflux and inhibits foam cell formation, whereas nuclear receptor corepressor 1 (NCOR1) exerts protective effects by restraining the pro‐atherogenic activity of PPARγ [33]. In addition, the natural product convallatoxin has been reported to attenuate AS progression by promoting M2 macrophage polarization through the PPARγ–integrin αvβ5 signaling pathway [22]. Consistent with these findings, ox‐LDL exposure significantly reduced PPARα and PPARγ protein expression in this study, whereas BDH1 overexpression effectively reversed these changes. Notably, the PPAR pathway‐specific inhibitor GW9662 markedly attenuated the beneficial effects of BDH1 overexpression on cell viability, lipid accumulation, and inflammatory responses, shifting these parameters toward the ox‐LDL‐induced injury phenotype. The protective effects exerted by BDH1 are, at least partially, channeled through the activation of the PPAR signaling pathway, as evidenced by these findings.

However, a number of shortcomings associated with this study deserve to be formally noted. First, the inhibitors used in this study are not site‐specific. MB‐3 inhibits GCN5‐mediated acetylation globally, and ACSS2i affects multiple acylation pathways, including acetylation, crotonylation, and lactylation, in addition to β‐hydroxybutyrylation. Therefore, the rescue experiments using these inhibitors cannot definitively attribute the observed phenotypic changes to H3K9ac or H3K9bhb alone. It remains possible that other histone sites or non‐histone proteins also contribute to the BDH1‐mediated effects. Second, the present study relied on global histone modification analysis by Western blot, which cannot distinguish locus‐specific changes. Third, the animal model was confined to ApoE^−^/^−^ mice, and the general applicability of BDH1 in other AS models has not been verified; the precise role of BDH1 in atherosclerotic plaques in vivo requires further validation using conditional gene knockout or overexpression animal models. Future studies employing site‐specific histone mutants, chromatin immunoprecipitation followed by sequencing (ChIP‐seq), or locus‐specific epigenetic editing will be necessary to establish direct causal relationships between specific histone acylation marks and BDH1‐mediated macrophage phenotypes.

To summarize, this investigation furnishes robust evidence that BDH1 is downregulated in atherosclerotic lesions and that it effectively attenuates ox‐LDL‐induced macrophage lipid accumulation and inflammatory responses at least in part by modulating histone acylation, including H3K9ac and H3K9bhb, and by activating the PPAR signaling pathway. These findings not only advance our comprehension of the epigenetic mechanisms underlying AS, but also furnish experimental proof validating BDH1 as a promising therapeutic target for the disease. Going forward, research priorities should be directed toward assessing the clinical applicability of BDH1 and devising targeted interventions against it, thus opening up novel avenues for AS prevention and treatment.

## Author Contributions

**Yu Wang:** investigation, methodology, formal analysis, data curation, writing – review and editing, writing – original draft. **Yi Wang:** methodology, formal analysis, writing – review and editing. **Qinghong Liu:** formal analysis, writing – review and editing. **Mao Tian:** validation, writing – review and editing. **Bin Wang:** methodology, writing – review and editing. **Qiang Lin:** formal analysis, writing – review and editing. **Shuiying Huang:** formal analysis, writing – review and editing.

## Ethics Statement

The experiments conformed to the Guide for the Care and Use of Laboratory Animals. Animal study has been approved by the Animal Ethics Committee of Southwest Medical University (No. 20221109‐012). All methods are reported in accordance with ARRIVE guidelines.

## Conflicts of Interest

The authors declare no conflicts of interest.

## Clinical Trial Number

Not applicable.

## Supporting information


Supporting File

## Data Availability

The data that support the findings of this study are available from the corresponding author upon reasonable request.
