# Supplementary material for: BDH1 Attenuates Macrophage Foam Cell Formation and Inflammation in Atherosclerosis Via Modulating Histone H3K9 Acylation Balance and Activating PPAR Signaling
Source: J Biochem Mol Toxicol. 2026 Oct 25;40(11):e71141. doi: 10.1002/jbt.71141 (PMC13695998; doi:10.1002/jbt.71141)
Supplement: Supplementary file 1 [file JBT-40-e71141-s001.docx]

**Supplementary Table 1 Primer sequences for RT-qPCR**

| **Primer name** | **Sequences (5’-3’)** |
| --- | --- |
| GAPDH-F | CTCATGACCACAGTCCATGC |
| GAPDH-R | TTCAGCTCTGGGATGACCTT |
| BDH1-F | AACTTGGTGCCACGATGCTA |
| BDH1-R | GCTGATGTTAACGACGCGAC |
